# Realist synthesis of co-production of services in a local authority context: what works, for whom, how and in what circumstances?

**DOI:** 10.1186/s12961-026-01522-0

**Published:** 2026-09-12

**Authors:** Andrew Harding, Reuben Tete Larbi, Aviel Lowndes, Paul Marshall, Adam Mars, Michaela Goodridge, Pauline Wigglesworth

**Affiliations:** 1 Division of Health Research, Faculty of Health and Medicine, Lancaster University, Lancaster, United Kingdom; 2 Blackpool Council, Blackpool, United Kingdom; 3 Institute of Population Health, Faculty of Health and Life Sciences, University of Liverpool, Liverpool, United Kingdom

## Abstract

**Background:**

Co-production is increasingly promoted as a means of improving the design and delivery of local authority services, yet evidence on how and why it works remains limited. This realist synthesis explored how co-production operates within complex local authority systems, identifying the circumstances and mechanisms that enable or constrain meaningful involvement of stakeholders in co-production processes and sustained change to ensure that services are co-produced with professionals and those with lived experience.

**Methods:**

Scoping searches identified key literature on co-production related to a local authority context to develop initial programme theories. Context–mechanism–outcome (CMO) configurations were constructed as “if–then–because” statements under four themes: equity and inclusion; collaboration and systems change; ethical and sustainable engagement and measurement and evaluation. These were refined through an analysis of 25 sources from searches across Scopus, CINAHL, Academic Search Ultimate, Web of Science and SocINDEX. Further refinement took place in two workshops with six participants with lived experience and four local authority officers. Analysis was thematic, retroductive and iterative to promote critical engagement between the research team, the literature and workshop participants.

**Results:**

Nine CMOs and an overarching programme theory illustrate how relational, structural, ethical and evaluative mechanisms interact to support authentic co-production. First, trust emerged as a central mechanism for engagement, built through culturally competent, inclusive and power-sharing practices. Second, sustainable co-production depended on visible leadership commitment, long-term investment and opportunities for skill development and emotional support. Third, ethical integrity and trauma-informed facilitation were essential for creating safe spaces where people felt respected and brave enough to share their vulnerability. Fourth, cross-sector collaboration and systems thinking enabled shared purpose and accountability across organizational boundaries. Finally, participatory and creative approaches to evaluation strengthened trust, demonstrated impact and promoted continuous learning. Concepts such as trust, power-sharing and leadership commitment operated recursively across different contexts and at varying system levels.

**Conclusions:**

Authentic co-production in local authority systems depends on aligning relational trust, ethical practice and structural support within learning cultures that value lived experience. This synthesis advances theoretical understanding and offers practical guidance for embedding equitable, sustainable collaboration into the everyday culture and governance of local authorities.

## Introduction

In England, local authorities are statutory local government organizations and public bodies responsible for delivering a wide range of services, including housing, education, aspects of public health and social care. Given their broad remit and close connection to communities, they are well placed to draw on lived experience to influence the upstream determinants of health and reduce inequalities. The people that local authorities directly engage with include (but are not limited to) children with special educational needs and disabilities (SEND), children in care and individuals or families with social care or housing needs [1, 2]. There is a great deal of evidence that local authority services are challenging for residents and community members to interact and engage with. For example, both parents with experience of local authority Children’s Social Care (CSC) and professionals often find the CSC system complex to navigate [3, 4]. Services that are not co-designed with users and have bureaucratic processes and language and lack appropriate human support systems leads to mistrust and poor uptake [5]. Another example is the process of child separation as a safeguarding intervention, which is often described as traumatizing for parents and further compounds existing vulnerabilities of those involved [6]. Services designed by professionals for so-called vulnerable groups have the tendency to be ill-equipped to meet the needs of intended beneficiaries [7].

Co-production is an established route to public service reform that enables those who engage with services to contribute to and be partners in the design and delivery of services [8]. The concept of co-production is not new [9, 10] and a wide variety of terms now exist that refer to the role of citizens in the design and delivery of public services. These terms include co-creation, co-design and co-delivery. The role of lived experience in shaping the delivery of services through participation has been widely promoted as a route to widespread and deep system reform in areas where local authorities have statutory obligations [11] as it seeks to deliver more inclusive, equitable and trusted services [12, 13]. In a local authority context, we define co-production as:

delivering public services in an equal and reciprocal relationship between professionals, people using services, their families and their neighbours. Where activities are co-produced in this way, both services and neighbourhoods become far more effective agents of change [14].

Although not a new concept and despite its potential and aim to promote inclusive, equitable and trusted service delivery, a statement from nearly a decade ago that the evidence base is relatively weak in a local authority context still has relevance [15]. Furthermore, there are clear gaps in the context of how co-production is operationalized and “works” (and does not “work”) in the context of socio-economic deprivation and inequalities where there will be unique service related challenges (3–7; 12,13). Most of the current literature has focussed on the conceptual framing or descriptive explanations and outcomes of initiatives rather than unpacking the conditions, mechanisms and contextual factors that shape outcomes. Specifically, some scholars have tried to create an analytical and conceptual boundary by identifying key principles of co-production [16–18], such as requiring and seeking to restore trust in public services [19] and what co-production is not [20, 21]. Other scholars have situated co-production within the broader participatory methodologies and highlighted its relevance in empowering communities to have a voice in public decision [20, 22, 23]. Others highlight the “dark” side of co-production [24–26], the challenges of co-producing services [21], why citizens engage in co-production [27–29] and building institutional capacity for co-producing services [13, 30–32]. Crompton [33] points to the professional and “elite” narrative of co-production being one where there is assumed to be equality of stakeholder voice, but also that gaps exist in the understanding of what works when co-producing services, for whom, under what circumstances and through what mechanisms [33]. This gap is particularly evident and important to explore in the complex, hierarchical and bureaucratic structures of local authorities [34, 35].

Foundational and theory-driven approaches such as realist synthesis can begin to provide a robust methodological response to this gap [15]. Unlike traditional systematic reviews that summarize evidence on a topic or subject area, the theory-driven and explanatory strength of the realist approach enhances its power to identify underlying generative mechanisms that produce outcomes in specific contexts [36–38]. Given that the aim is to engage with the complexity of social programmes, realist synthesis allows for the inclusion of a broad range of evidence. This approach is thus well suited to the study of co-production in a local authority context where there exists a wealth of empirical and nonempirical work and diverse stakeholders, structural constraints and competing priorities interact.

Through a focus on the interplay between context, mechanisms and outcomes (CMOs), realist synthesis can generate nuanced insights into the conditions that foster or hinder meaningful co-production [37, 39]. This paper presents a realist synthesis to further evidence-informed understanding of co-production that foregrounds issues of equity, inclusion, ethics, sustainability and systems-level change. By drawing on the literature and insights from stakeholders we synthesize and refine theoretical propositions about co-production in a local authority context. In doing so, the paper addresses the limitations of existing studies that overlook the unique contextual complexities of local government striving to embed co-production in ways that are both meaningful and impactful.

## Methods

Realist synthesis is a theory-driven approach to understanding causal processes based on a realist philosophy of science [37]. The central aim of the realist synthesis is to develop and refine programme theory in the form of context, mechanism and outcome configurations (CMOc) that are middle range and sufficiently abstract, and in the case of this synthesis, to be applicable to co-production approaches within the broader scope of local authority settings. This paper follows the established realist synthesis reporting standards and publication guidelines [37, 39, 40]. Our review was registered on the International Prospective Register of Systematic Reviews (PROSPERO; CRD420251077691). Given that we also held stakeholder workshops to further refine the programme theory, ethical approval was sought and attained from the Faculty of Health and Medicine Ethics Committee [Reference: FHM-2025-5527-RECR-2].

### Stage 1: realist review

The review is driven by four key questions:

1. What are the outcomes for service users that result from co-production in local authorities?
2. For whom does co-production work in local authorities?
3. What contextual factors (such as resources) are necessary for co-production in local authorities?
4. What mechanisms explain the impact of co-production on individuals and services in local authorities?

### Developing initial programme theory through scoping searches, key papers and research team discussion

We began by developing an initial programme theory (IPT) to guide the realist synthesis. This was informed by an iterative process combining scoping searches, review of key papers and structured discussions within the research team and with stakeholders.

### Scoping searches and key literature

We used known key papers and conducted preliminary scoping searches across bibliographic databases (e.g. MEDLINE, Google scholar, Social Care Online) and grey literature to identify work on co-production relevant to a local authority context. Key papers were selected on the basis of their conceptual depth, empirical relevance and resonance with our focus on local authority practice which broadly included sources relating to health and social care. For example, one of these was an existing rapid realist review of co-production in youth mental health services [41]. Collectively, the key papers provided candidate explanations for how and why co-production may or may not work within local authority systems [12, 13, 42–50].

### Development of themes

Drawing on these sources, we inductively developed four thematic domains around which early programme theories could be organized:

- Equity and inclusion – who is involved, how participation is enabled and how trust is built.
- Collaboration and systems change – the extent to which co-production helps break down silos and facilitates cross-sector working.
- Ethical and sustainable engagement – ensuring integrity, reciprocity and longevity of co-production activities.
- Measurement and evaluation – capturing impact, demonstrating value and refining practice.

### From themes to candidate theories

Within each theme, the research team used abductive and retroductive logic to generate candidate theories in the form of provisional “if–then–because” statements based on the sources located in the scoping searches [51]. Rival theories were also deliberately developed to capture alternative explanations. An example of a candidate theory can be found in Appendix 1. This process produced a set of draft programme theories, which were subsequently tested and refined through engaging with relevant literature and in stakeholder workshops.

### Search strategy

In May 2025, searches were conducted across five databases – Scopus, CINAL, Academic Search Ultimate, Web of science and SocINDEX. The selection of databases and development search strategy were done in consultation with information specialists with expertise in the subject (an example search string from Scopus is included in Appendix 2).

### Inclusion criteria

Sources were included in the synthesis if they were:

(i) Empirical studies or grey literature
(ii) Focussed on co-production, co-creation, co-delivery, co-design in a health social care or local authority context
(iii) Available in English

### Deduplication and screening

**Fig. 1:**
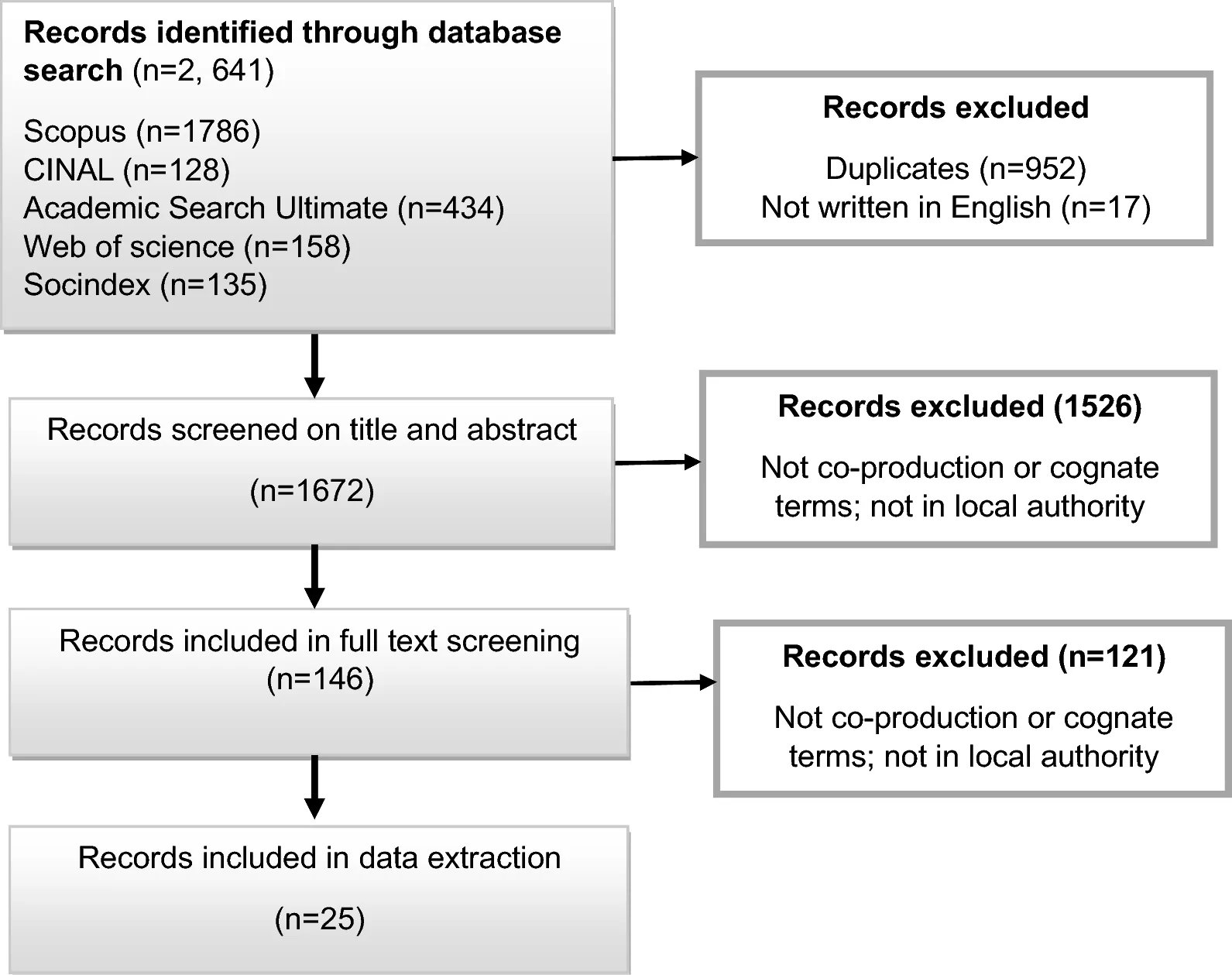
PRISMA flow diagram

All records were exported into Endnote and duplicate records were removed. The records were then exported into Rayyan. Titles and abstracts were screened followed by full-text screening of potentially relevant sources. A.H. and R.L. screened 10% of records and conflicts in included/excluded paper were discussed to ensure a consistent screening process. The process of searching and screening papers is summarized in Fig. 1.

### Relevance and quality appraisal

Included studies were appraised using the criteria of relevance, richness and rigour, as recommended by Dada et al. [52]. Relevance was assessed in terms of whether a study offered insights into contexts, mechanisms or outcomes that could inform programme theory development. Richness referred to the extent to which a study provided explanatory depth, for example, through detailed descriptions of processes, participant perspectives or accounts of how and why an intervention worked (or did not). Rigour concerned the credibility and trustworthiness of the evidence, including the appropriateness of study design, transparency in data collection and analysis and the extent to which limitations were acknowledged or mitigated.

Each included paper was read in full to gain familiarity with its contribution and appraised on these three dimensions. Studies were not excluded based on methodological quality alone; instead, appraisal informed judgements about the relative weight of evidence in theory-building. Studies assessed as highly relevant and rich were prioritized in the synthesis, even where rigour was moderate, whilst those with weaker relevance or limited explanatory value were used more cautiously. The process was iterative, with studies revisited as the programme theory developed, allowing for sensitivity testing of appraisal judgements as new mechanisms and insights emerged [37, 53].

### Data extraction and analysis

An Excel template was developed to support data extraction on the characteristics of the included sources and reported co-production approaches and research methods (if appropriate). In addition, we used the realist review appraisal form developed by Molitor et al. [54] to guide the extraction of data on contexts (C), mechanisms (M) and outcomes (O). This approach supported the articulation of C + M = O connections, as well as the identification of potential rival theories. We applied thematic analysis to identify recurrent, demi-regularities and salient patterns across the included papers [55]. Once extracted, the data were organized in a three-column table, with columns for context, mechanism and outcome of each IPT. Using retroductive reasoning and discussion amongst the research team, these steps provided an iterative process that facilitated the analysis and synthesis process [56]. On the conclusion of this stage of refine, the programme theories were then further refined in two workshops with key stakeholders.

#### Stage 2: workshops with key stakeholders

##### Participants and sampling

We purposively recruited two stakeholder groups to get insights from a lived experience and systems or organizational perspective:

1. People with lived experience, or experts by experience who were also employed in roles relating to co-production within the local authority context;
2. Council officers / practitioners/decision-makers including managers, frontline practitioners and commissioners.

We sought variation in role, degree of experience, service area and exposure to co-production to ensure a diversity of perspectives on contexts, mechanisms and outcomes [37, 38]. The expert by experience and professional stakeholder groups took place separately to mitigate power dynamics and create safe space spaces for participants [57]. The workshops were facilitated by an experienced and trained researcher in realist approaches (A.H.) and a practitioner trained in trauma informed practice (A.L.).

### Materials

For each IPT, we developed “theory cards” summarizing the key CMO propositions as “if–then–because” statements to make programme theories explicit and open to modification [37, 58]. Each theory was presented and critical feedback and discussion, following an iterative cycle of stakeholder input, theory refinement, and negotiation. This reflected a process of “theory adjudication”, whereby emerging explanations were critically considered and refined in light of stakeholder knowledge and perspectives [38, 58].

### Data analysis and refined programme theory

All workshop sessions were audio-recorded and facilitators captured changes on annotated theory cards and notes. Immediately following each workshop, the team compared and discussed their notes, coding suggested changes by CMO component and noting contested or rival versions. We then transcribed and analysed the transcripts engaging in abductive and retroductive reasoning to reconcile stakeholder inputs and published evidence, revising IPTs accordingly [56].

## Results

### Overview of included literature

A total of 25 papers were included in the synthesis, representing research from 11 countries. The largest proportion were conducted in the United Kingdom (*n* = 10) [13, 15, 33, 59–65], then two from Denmark [43, 66], two from Italy [67, 68] and three from the United States [69–71]. Single papers came from Sweden [72], Japan [73], Israel [74] and Turkey [75], and four papers provided cross-European contexts [19, 76–78]. In terms of type of evidence, 22 were peer reviewed journal articles, 2 were book chapters, and 1 was conference proceeding.

Regarding study design, 10 papers used qualitative case study design exploring co-production within local government contexts [33, 60, 61, 63, 64, 66, 68, 72, 73, 77]. Four papers were quantitative [19, 59, 71, 74], whilst two were mixed methods integrating qualitative and quantitative data [13, 67]. Seven papers were primarily theoretical or conceptual developing models or frameworks on co-production [15, 43, 63, 69, 75, 76, 78].

These papers examined population and contexts including citizens and service users in public services [19], groups such as young people [60], caregivers [68], people with dementia [62] and learning difficulty [66] and care leavers [72], as well as public-sector staff and community actors involved in co-production [33, 63, 65]. Overall, this literature spans a diverse range of contexts, methodologies and countries, providing comprehensive overview of research on co-production in local government contexts.

Workshops

Six lived experience members took part in their workshop and this was followed by a workshop involving four council officers. To maintain a level of accessibility, the lived experience members’ preference was to keep the format of the “if–then–because” statement as opposed to presenting CMOs, which were thought to be complex and relatively inaccessible. On this basis, we present the refined programme theory in this format, though with “context”, “mechanism” and “outcome” presented in brackets. In total, our synthesis presents nine CMOs with cross-cutting factors and mechanisms and an overarching programme theory.

## Enhancing equity in co-production

Enhancing equity in co-production encompasses who is involved, how participation is enabled and building trust and connections [13, 73]. In terms of who is involved, Gheduzzi et al. [67] highlight that the unique characteristics of individuals involved in co-production reflects the users of services being produced, and there should be deliberate efforts to include those who have experienced barriers or perceived bad experiences. Whereas the organizational aspects of co-production such as having a culture that encourages inclusion and involvement of citizens are similar regardless of the sector, the procedural aspects such as how citizens are involved tend to vary by sector. Particularly, the often complex and challenging circumstances experienced by people in health and social care require professionals to dedicate more time to building trust and creating an enabling environment for those who may not initially feel confident or empowered to participate in co-production activities. Trust is commonly theorized as a pivotal mechanism in co-production [71]. As Fledderus et al. [19] argues, different forms of trust may be fostered through co-production, each operating through specific mechanisms and contingent on particular conditions. In our synthesis, trust is not treated as a background condition but as an outcome of relational interactional processes where meaningful power sharing occurs, and once established, becomes a mechanism itself for sustaining and deepening co-production that can address inequity and unfairness in health and social care through prolonged and honest involvement. Participants in the workshop commented:

...it’s about genuinely sharing power. So there’s something about genuinely handing over the control, the power to the people who are receiving services to design those services and have a meaningful, impactful voice in the design of those services... And then it’s then the result of those services, they become more bespoke, personal and specific, reflect people’s own actual particular needs (council officer, children’s social care).

The lived experience group felt power dynamics always exist between professionals and lived experience groups, and asked for the need to invest in relationship building:

...it’s about the time we put in before to build relationships ...If there’s not enough time for that, which often when you’re doing co-production work, there isn’t. That’s why I think it then falls apart to this alternative (expert by experience, co-production team).

...we’ll make people feel valued, but like we say, we should be making them feel valued and develop a sense of warmth (council officer, special educational needs).

...they’re more willing to stay involved, speak up about what they need. I’d say share their lived experience honestly (council officer, children’s social care).

In terms of conceptualizing broad outcomes of co-production, Uster et al. [74] reports a positive relationship between the extent to which citizens co-produce in local service delivery process and the effectiveness of services. Co-production has been used as a tool to empower citizens to contribute meaningfully to the design and implementation of services that they rely on [75, 78]. On this basis, our analysis proposes the following CMO:

*If people face barriers such as complicated rules, past bad experiences or services that do not reflect their culture or needs, they are less likely to get involved (context).*

*However, if co-production cultivates relationships and shares power meaningfully, people feel valued, confident and develop a sense of warmth, belonging and trust (mechanism).*

*Then individuals, families and communities are willing to stay involved, share their lived experience honestly and help shape services that work for everyone, reducing unfairness in health and social care (outcome).*

Our synthesis also revealed circumstances where trust does not manifest or erodes if co-production is undertaken poorly, not seriously or is tokenistic. In the literature, factors such as perceptions of processes driven by a predetermined management agenda (and lived experience not driving the agenda from the start), a lack of transparency and poor facilitation where people are unable to express themselves can disenfranchise lived experience participation and ensure hierarchies are not challenged and that professional voices are dominant [33, 77]. As stated in our workshops, “you need a commitment from the top….” (council officer, children’s social care), and traditional organizational hierarchies were cited as a barrier to co-production:

…loads of public sector organizations are so obsessed with the vertical structures with the hierarchy of people… where you kind of, you can’t do anything unless that person above you says that’s okay. And that’s hugely inhibitive to a lot of like co-productive work and allowing people freedom and autonomy and trust to actually make decisions and be part of things (ibid).

Others highlighted how poor facilitation can be a barrier to meaningful co-production:

if you don’t have effective facilitators it doesn't work either. I think like you say you need that someone who’s going to bring it back, make it meaningful, kind of stop people saying no that’s a solution, we’re not, we’re on week three and we’re not there yet (council officer, child specific programme).

Participants of the lived experience group highlighted the value of being involved from the outset of programmes as opposed to one-off tokenistic participation as:

I think the other thing for this is it’s about, for us as well, it’s about when they get us involved, how long we’re involved for. So just bringing us in for one, let’s say one focus group or a conference or something, just to talk about our experience, that comes back to being tokenistic, doesn’t it? Whereas if we’re involved from the beginning, like the planning, the talks about what we all want to achieve, solutions, barriers, that kind of thing, all the way through to completion, I think that then becomes less tokenistic (expert by experience, co-production team).

Another expressed concerns about co-production not resulting in intended outcomes when it begins but is not followed through:

….everybody’s excited and wants to work together and wants to do something productively. But again, because it’s not followed through, everybody’s not been involved from the beginning. They go off to their own roles, which then takes up quite a lot of time. Things aren’t happening, so no, it doesn’t happen. So you still feel like your voice has been, you know, useless in that because it’s not made any changes (expert by experience, co-production team).

On this basis our analysis proposes a rival theory where, in these circumstances, trust does not manifest because processes are tokenistic:

*If organizations try to involve communities but don’t really commit – for example, people aren’t involved from the start, leaders do not support it, staff are not trained to facilitate properly or old hierarchies get in the way – people’s involvement ends up being tokenistic (context).*

*Then trust in services falls, people withdraw and services stay out of touch with the real needs of the communities they are supposed to serve (outcome).*

*Because people see this as just a “tick-box” exercise rather than real change, they feel frustrated, sceptical and give up taking part (mechanism).*

## Meaningful systems change

In the workshops it was stated how people can be passed around “the system” and that this can be retraumatizing for people who have to constantly explain themselves and leads to a further perceived loss in status (council officer, social work). This can create a challenge to get people involved to work towards meaningful systems change and involving those who have been retraumatized is a challenge, as it extenuates power dynamics, and this can be a challenge for partnership working in co-production.

In two case studies of co-production within National Health Service (NHS) and local government, Farr [63] highlights how co-production must explicitly address power dynamics in their facilitation, management and coordination, recognizing that they unfold within a complex web of psychological, social, cultural and institutional interactions. This was reinforced in one of the workshops:

Regardless of who organizes the room, who’s in the space, there’s always going to be a power dynamic between lived experience and practitioners. Always…Rebalance that power where we see each other as human beings (expert by experience, co-production team).

Power and status differences, if not addressed through facilitation, can lead to those with lived experience becoming passive [33, 68, 73]. Respectful and meaningful structured partnerships were stated by workshop participants as a means by which power dynamics can be challenged so that people feel their voices and experiences are valued.

...the kind of solutions are relationship-based, and the skills with a lot of this stuff is around the relationships, and exactly what you’re describing for taking the lanyards off, often like the first sessions in co-production are just... learning to love each other, actually just kind of getting on with each other (council officer, children’s social care)

Such is the value of this to the culture of organizations, those with lived experience felt strongly that “cross – sector collaboration and systems change” was not an adequate reflection of what co-production can achieve within a local authority setting. Instead, there was a call instead to emphasize the importance of “meaningful systems change” through respectful and meaningful structured partnerships as a means to developing a collaborative culture to breakdown barriers between organizations:

*If health, education and social problems are tackled separately by schools, doctors, councils and community groups without working together, people end up falling through the cracks and repeating issues can retraumatize and lead to further perceived loss of status (context).*

*However, if co-production “gets the right people in the space” it helps bring these groups together in meaningful structured partnerships, it shows a real commitment to working jointly. Even with different roles, status and power dynamics, this builds respect between all people involved to feel their voices and experiences are genuinely valued, which motivates them to get involved (mechanism).*

*Then services develop a structural collaborative culture, break down barriers between organizations and make sure communities get the support they actually need (outcome).*

In contrast, workshop participants outlined how factors such as competing priorities, unclear aims and a lack of common purpose can lead to professionals being defensive because their “expertise” is being challenged, and how this competitive aspect (as opposed to meaningful partnerships) can derail collaborative working:

…you have to have that common thing that you’re working towards… (council officer, child specific programme).

…when it doesn’t work and we’re kind of being defensive and we’re kind of like almost competing with each other rather than actually that kind of, yeah, that collaborative spiral (council officer, children’s social care).

*If people try to work together but have competing priorities, unclear aims, goals and roles, or not enough resources, collaboration does not run smoothly (context).*

*Then partnerships and relationships start to break down, and organizations slip back into working in silos – meaning real systems change never happens (outcome).*

*Tensions arise because professionals may feel their expertise is being challenged, there is a lack of shared understanding or agencies may be unwilling to share power with communities (mechanism).*

## Ensuring co-production is ethical

A lack of trust in institutions can lead people to perceive that involvement will be tokenistic, particularly when previous engagement experiences have been extractive or symbolic [12, 59]. However, establishing strong ethical foundations – such as transparent consent, fair remuneration, trauma-informed practice and the creation of safe and inclusive spaces – can help to rebuild this trust. When participants perceive these safeguards as genuine, they are more willing to share personal experiences and emotional truths. Mortensen and Needham [66] suggest that when staff “establish norms of emotional closeness with service users this has a humanizing effect which dismantles barriers” (p. 1273). In our analysis, emotional closeness is not incidental but a deliberate outcome of ethical practice that creates the conditions for people to feel brave enough to be vulnerable. This form of relational courage is central to developing meaningful and authentic partnerships within co-production. Participants in our workshops described how emotional closeness, courage and care emerge through ethical practice:

So how do you support people and understand that that has a real impact and that their contribution is not just valid but worth paying for and needs supporting properly too (council officer, children’s social care).

So they have the courage to be vulnerable and be brave (council officer, social work).

If you go in and understand that actually it’s a relationship and you sharing something about yourself and being able to be vulnerable, to be able to kind of like connect in a genuine way and build that relationship, there’s an intimacy in that work… when you are able to be vulnerable, able to share, be intimate, form relationships (council officer, children’s social care).

With regards to the creation of safe and inclusive spaces, experts by experience noted that:

Yeah, yeah. So, if people worry that they’re invited to be tokenistic or that sharing their experiences could leave them exposed or unheard, they may be reluctant to take part.

… because some people have been through traumas and it does affect them. And even if they have shared the story, it’s like you need to know if they’re all right before you send them on the way.

The experts by experience group also introduced the concept of “brave space”, as providing the enabling space for people to be who they are and express themselves freely. This was premised on the notion that… “it’s just like reading the room to make sure everyone’s all right” and building relationships to know what individually people need, but you also have a brave group wellbeing. The concept of “brave space” was further articulated as:

I think if you give people the opportunities and the space to be who they are and express how they need to express without any kind of judgment or oppression or come back later on, it becomes a brave space, not safe.

Our analyses proposes the following CMO:

*If people worry that their involvement will just be tokenistic, are concerned nothing will change, or that sharing their experiences could leave them exposed or unheard, they may be reluctant to take part (context).*

*However, because co-production that puts strong ethical principles and safeguards in place – such as clear consent, shared decision-making, trauma-informed and responsive practice, appropriate remuneration, safe spaces for people to share and time to debrief – this makes people feel safe, brave enough to share their vulnerability, respected and valued (mechanism).*

*Then people are more likely to trust the process, join in fully and build meaningful and authentic relationships, creating long-term collaboration based on mutual respect (outcome).*

## A foundation for sustainable co-production

Maintaining co-production over time has consistently been identified as challenging, often due to practical and resource-related barriers but also because deeper cultural and structural shifts have not taken root [66, 69, 72, 76]. Without these foundations, professional hierarchies and role perceptions including tensions amongst collaborators can persist, limiting the influence of lived experience and curtailing the transformative potential of co-production. For co-production to be meaningful and sustainable, authors emphasize the importance of organizational and political commitment, including senior leadership endorsement, long-term funding and investment in those delivering and participating in co-production [60, 64, 65]. Olsson et al. [72] highlight the need for initial activities of the co-production process to include a discussion about uncertainties that may arise and expectations such as time limits. Regular check-ins would uncover issues that may arise but remain unspoken.

In our workshops, sustainability was described as dependent on both systemic support and individual development. Participants highlighted the need for ongoing emotional support, skills progression and paid roles for people with lived experience to sustain motivation and ensure involvement is both valued and impactful. Leadership commitment and cultural change were seen as equally vital for maintaining momentum and embedding co-production into the fabric of organizations rather than leaving it as a short-term project activity.

There needs to be infrastructure behind to look after those people afterwards... (council officer, special educational needs).

“I think almost the most important part is the cultural change, the political support here... That’s one of the biggest bits that makes these things sustainable” (council officer, children’s social care). “If you do not support them in the right way, give them the right progression routes, all that kind of stuff, that’s when you’re going to lose motivation and they’re going to stop being involved” (expert by experience, co-production team).

On this basis, our analysis proposes the following CMO:

*If co-production is supported through visible political and organizational commitment, long-term investment, community ownership and progressive pathways for skill development and emotional support (context).*

*Then lived and work experience becomes embedded into the system, and people develop pride, responsibility and commitment, sustaining co-production beyond initial project cycles (mechanism).*

*Because these conditions create a strong foundation for lasting involvement, services continue to be shaped and improved by the people who use and work within them (outcome).*

Whilst long-term commitment and organizational investment are central to sustaining co-production, short-term or poorly communicated efforts can have the opposite effect. When people’s contributions are not acted upon, or when the results of their involvement are not shared transparently, engagement can quickly lose momentum. Co-production, in these circumstances, risks becoming a performative exercise – replicating the very power imbalances it seeks to dismantle. Participants in our workshops described how limited follow-through, poor feedback loops and inconsistent leadership rapidly eroded motivation and trust, leaving people feeling undervalued and fatigued.

… you could take all the learning from that, you could go and make changes, but then if you don’t communicate back, they don’t see the change happening (council officer, child specific programme).

It’s the lack of commitment… that definitely doesn’t act on what they say, because that’s the tokenistic bit, isn’t it? (council officer, child specific programme).

These reflections highlight that sustainability is not only a question of resources but also of relational maintenance and visibility of impact. Communication and expectation management act as relational mechanisms that sustain trust and prevent emotional burnout. On this basis, our analysis proposes the following CMO:

*If people get involved in co-production but there is only short-term commitment, limited feedback or poor communication about outcomes and expectations are not managed (context).*

*Then those involved begin to feel worn out, let down and lose trust in the process (mechanism).*

*Because when their ideas are not visibly acted upon or translated into meaningful change, involvement feels tokenistic and emotionally draining, leading people to withdraw altogether (outcome).*

## The impact of co-production

Durose et al. [15] highlight the relative weakness of the evidence base on co-production, attributing this in part to policymakers’ preference for traditional hierarchies of evidence. Such preferences tend to privilege methods that emphasize generalizability and measurable outcome (such as effectiveness, resource mobilization or user satisfaction) whilst distancing evaluators from the co-production process itself. In doing so, they undervalue approaches that are closer in proximity to practice and better able to capture the richness, depth and lived experience that characterize co-production, but which are often less valued by key decision-makers [43]. Rippon [62] advocates for a focus on “how outcomes are produced” in addition to the current focus on the types of outcomes that result from co-production initiatives. A focus on the “how” would elucidate the role played by citizens in producing those outcomes. In our workshops, it was stated how using traditional key performance indicators (KPIs) would be unlikely to capture the evolving nature of co-productions impact on changing services and that participatory, collaborative and creative evaluation can also build trust because impact becomes more visible:

… if you only do the same KPIs, you’re only going to keep on doing the same things and you’re not going to improve… What about the impact on culture? (council officer, children’s social care).

What does it [KPI] actually mean? You can make a pretty graph, but what does that mean? It’s the context underneath, isn’t it? (council officer, social work).

Introducing more participatory, collaborative and creative ways of evaluating helps build trust that everyone’s impact. because it’s it is everyone’s isn’t it (council officer, social work).

On this basis, we propose the following CMOs based on our analyses:

*If co-production is only judged using narrow traditional performance measures, then cultural and systems change and the opportunity to capture transformational change gets overlooked (context).*

*However, introducing more qualitative, participatory, collaborative and creative ways of evaluation that capture people’s voice builds trust that their input makes a real difference, and gives everybody a clearer view of the long-term value of co-production (outcome).*

*Because these approaches enable people involved to better demonstrate impact, create a stronger evidence base, support ongoing improvement and ensure that co-production continues to evolve and change systems in meaningful ways (mechanism).*

*If co-production is only measured by short-term numbers such as costs or quick results, the deeper benefits – such as stronger relationships and long-term change – are ignored (context).*

*Then people taking part start to feel their efforts do not really matter and lose faith in the process. Over time, motivation fades and co-production gets pushed aside in favour of projects that can show quick, easy wins (outcome).*

*Because without meaningful ways to measure impact, it is hard to prove and difficult to see the real value of co-production, thus decision-makers stop prioritizing it (mechanism).*

We now discuss the CMOs and mechanisms to identify patterns to advance the synthesis and to present an overarching programme theory.

### Cross-cutting and interconnections between mechanisms and developing an overarching programme theory

Many of the factors and mechanisms are cross-cutting and have resonance across the CMOs. Therefore, we propose that the mechanisms identified in this review did not operate in isolation but interacted dynamically. Trust, for example, was both fostered by ethical practice and sustained through long-term structural investment. Power-sharing and clarity reinforced each other, whilst participatory evaluation helped maintain transparency and legitimacy. Disruptions in any one domain – such as leadership turnover, poor communication or inadequate funding – is considered as potentially undermining the others, highlighting the fragile nature of co-production ecosystems. The thematic map developed in this study illustrates these interdependencies, positioning authentic, sustainable co-production at the centre of five interconnected clusters: trust; power, clarity and relational equity; ethical and emotional dimensions; structural and organizational enablers; and reflexivity and evaluative learning. This framing underscores that co-production succeeds not through isolated actions but through the alignment of these interrelated mechanisms and processes.

## Discussion

This realist synthesis engaged with literature and key stakeholders to understand how, why and under what conditions co-production contributes to improved design and delivery of local authority services. By synthesizing evidence through a realist lens, we identified a set of context–mechanism–outcome (CMO) configurations – expressed as “if–then–because” statements – that illustrate the relational, structural, ethical and evaluative processes underpinning effective co-production. The findings are consistent with existing research and literature, but also extend conceptual understanding. We develop theoretically grounded explanations with key stakeholder insight to provide a more advanced conceptual causal understanding of co-production which to date, as we outline below, has either tended to highlight the importance of some of these factors or present it in aspirational terms. The synthesis emphasizes trust; power, clarity and relational equity; ethical and emotional dimensions; structural and organizational enablers; and reflexivity and evaluative learning as key mechanisms that sustain authentic collaboration.

### Positioning within the literature

Our findings build directly on and extend the work of Brix et al. [43], van der Graaf et al. [13] and Jones et al. [41], each of whom highlight the structural and relational conditions that enable meaningful co-production. Brix et al. [43] developed a programme theory of co-production that emphasized trust, ownership and citizen participation as mechanisms linking governance structures to improved service outcomes. Van der Graaf et al. [13] identified adaptive governance, evidence codification and collective reflection spaces as key enablers within local government, whilst Jones et al. [41] focussed on the role of trust, power-sharing and organizational culture in shaping collaborative practice in youth services. Our work builds on these foundations by synthesizing them, along with other secondary and primary sources, to a UK local authority context – an environment characterized by heightened complexity, statutory constraints and power dynamics [13, 34, 35]. We deepen these frameworks by unpacking the reasoning and emotional processes – such as feelings of warmth, belonging or disillusionment – that underpin peoples’ responses to when co-production is done well and not so well, thereby adding explanatory depth to how trust and power operate in practice. We also introduce rival pathways to capture why co-production may fail, highlighting mechanisms such as burnout, ethical lapses or unmet expectations. In addition, we differentiate between the ethical and sustainability strands implicit in Brix et al.’s [43] theory and propose that emotional safety, appropriate remuneration and long-term investment each play distinct and essential roles. Finally, by integrating participatory evaluation and reflexivity as active mechanisms that reinforce legitimacy and learning, our work links the structural focus of Brix [43] and van der Graaf [13] with the relational insights of Jones et al. [41] to propose a realist, mechanism-based framework for authentic, sustainable co-production in a local authority context.

**Fig. 2:**
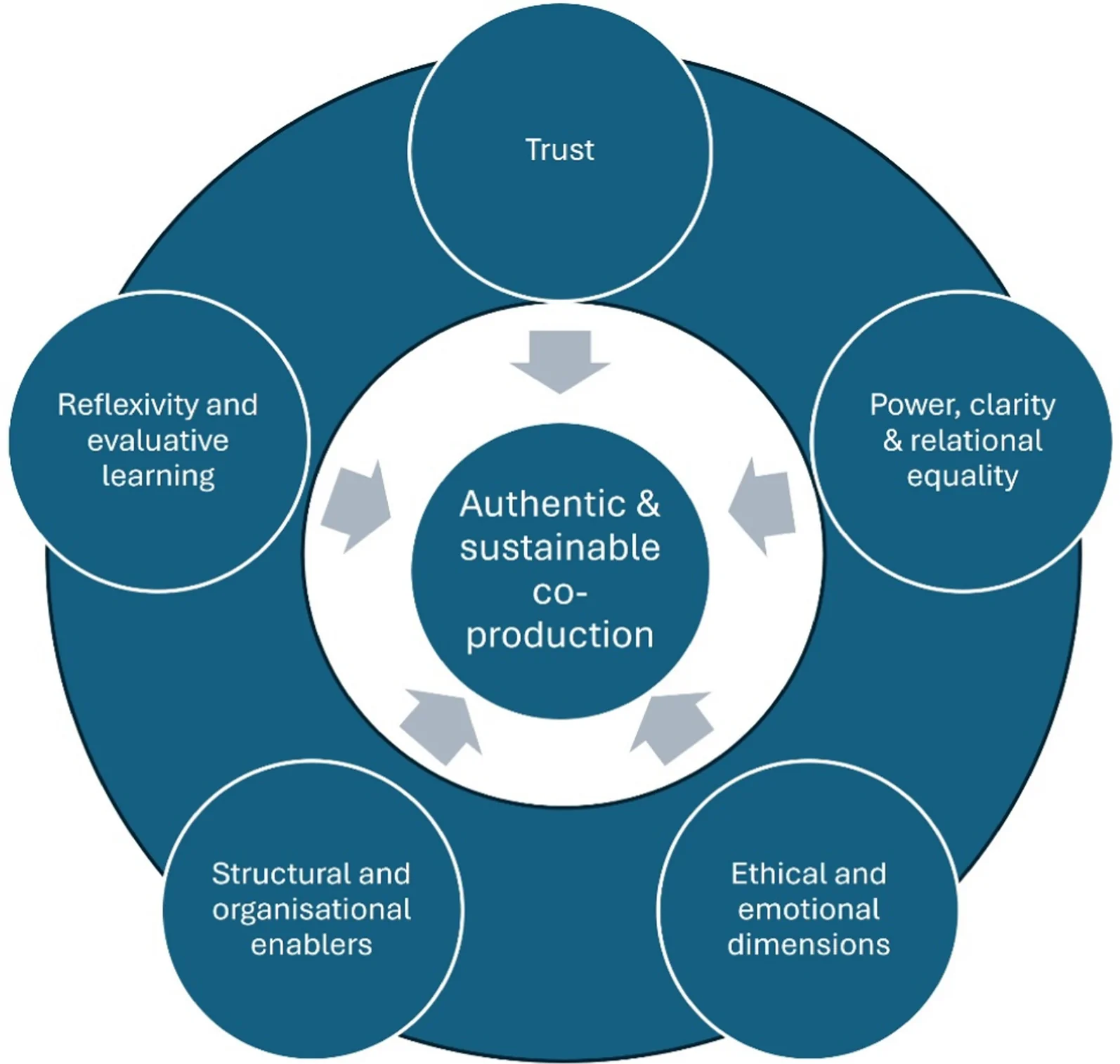
The interconnected mechanisms of co-production

We further developed our synthesis and considered how many of the factors and mechanisms were cross-cutting given their application in multiple CMOs. On this basis we propose an overarching programme theory shown in Fig. 2, which positions co-production as a dynamic, multilayered system shaped by the interaction of relational, structural, ethical and learning mechanisms. As above in relation to the nine CMOs, the overarching programme theory integrates the adaptive governance and reflection spaces highlighted by van der Graaf et al. [13], the trust and shared power mechanisms identified by Fledderus [12] and Jones et al. [41] and the generic model of ownership and participation advanced by Brix et al. [43], whilst extending them through a deeper focus on people’s reasoning and emotion. It recognizes that trust is both a precondition and an emergent outcome of co-production, sustained through ethical integrity, long-term investment and iterative learning. Our programme theory accounts for how co-production succeeds or fails depending on how these mechanisms align within specific organizational and community contexts. This provides a unifying framework that not only consolidates prior theoretical developments, but also offers a practical tool for identifying the conditions under which co-production could generate authentic, lasting change.

### Implications for policy, practice and further research

From a practice perspective, these findings emphasize the need for local authorities to invest not only in participation structures but also in the conditions that make participation meaningful. This includes providing facilitation training, supporting emotional wellbeing and embedding evaluation that values lived experience. Policymakers should prioritize long-term funding mechanisms that enable continuity and capacity-building, whilst leaders must model and champion power-sharing behaviours within their organizations. From a bottom-up perspective, co-production requires the fostering of lived experience and voice, but requires top-level buy in and culture change to make it meaningful and sustainable.

Methodologically, this synthesis reinforces the value of realist approaches for foundational and theory driven unpacking of the complexity of co-production in multilevel systems. The theories presented here, whilst based on a synthesis that included primary evidence, will benefit from further development from empirical research. We also echo Durose et al. [15], who suggest that evidence should capture the richness, depth and role of lived experience, but also that there is clear need to clearly and robustly link co-production to health and social outcomes of co-produced services to fully understand its value.

## Strengths and limitations

Applying the CMO framework to a complex, multilayered programme such as co-production in a local authority context presented both conceptual and practical challenges. The co-production processes examined here were dynamic, overlapping and likely influenced by multiple interacting factors that evolved over time. As a result, several CMOs in our analysis share similar underlying concepts – such as trust, power-sharing or leadership – which functioned across different contexts and at varying system levels. Rather than viewing this overlap as a limitation, we interpret it as reflective of the complexity of co-production and generative nature of these mechanisms: they are recurrent but differently activated depending on local conditions and relationships. This complexity required an iterative and interpretive approach to conceptualizing CMOs, where configurations were understood not as fixed chains of cause and effect but as heuristic tools for sense-making within an evolving system.

“Co-production”, along with related terms, are used in the literature to describe a wide range of activities. Although a realist synthesis can accommodate this diversity by drawing on evidence from varied programmes, it remained challenging to determine the precise nature of the co-production practices reported in many sources and studies. This variation highlights both the conceptual elasticity of the term [15] and the value of a realist approach for making sense of it. Given our aim to develop middle-range theoretical explanations that are broadly applicable, we anticipate that the findings of this synthesis will be relevant across local authority contexts – and potentially beyond – though ultimately its wider relevance can only be established with further research and or refinement in relation to other organizational contexts.

## Conclusions

This realist synthesis demonstrates that successful co-production in local authorities is achieved through the interplay of relational, structural, ethical and learning mechanisms that sustain trust and collaboration over time. Across the evidence analysed, co-production was most effective when power was genuinely shared, participants felt emotionally safe and valued and long-term investment supported continuity and growth. Trust emerged as both a product and driver of these processes – deepening engagement, strengthening relationships and enhancing the legitimacy of co-production as a means of system change. Our work builds on existing literature by showing how structural enablers interact with human relationships to generate authentic, sustainable co-production. Embedding these principles into everyday practice requires organizational commitment, transparent communication and participatory evaluation that demonstrates tangible impact. We propose that when these conditions align, co-production has the potential to move beyond rhetoric and become a transformative approach to designing and delivering local authority services that are equitable, responsive and trusted by the communities they serve.

## Data Availability

Data can be made available on request.

## Appendix 1: Example candidate theory

If people in marginalized communities face barriers such as complicated rules, past bad experiences or services that do not reflect their culture or needs, they are less likely to get involved. However, because co-production can make services more respectful, inclusive and human, people feel valued, confident and develop a sense of warmth, belonging and trust. Then they are more willing to stay involved, speak up about what they need and help shape services that work for everyone, reducing unfairness in health and social care.

Rival theory: If organizations try to involve communities but do not really commit – for example, leaders do not support it, staff are not trained to facilitate properly or old hierarchies get in the way – people’s involvement ends up being tokenistic. Then trust in services falls, people withdraw and services stay out of touch with the real needs of the communities they are supposed to serve. Because participants see this as just a “tick-box” exercise rather than real change, they feel frustrated, sceptical and give up taking part.

## Appendix 2

The search strategy combined terms relating to co-production with terms describing local authority contexts. The final search string used for Scopus search was: (Co-production OR Co-creation OR Co-design OR Co-delivery OR “Co production” OR “Co creation” OR “Co design” OR “Co delivery” OR Coproduction OR Cocreation OR Codesign OR Codelivery) AND (((local* OR county OR metro* OR municipal) W/3 (government*)) OR ((public OR municipal) W/3 (service*)))

This formulation ensured the inclusion of relevant synonyms and spelling variations, and the use of proximity operators (W/3) captured terms appearing within three terms of each other.

## Declarations

Ethical approval was granted by the Lancaster University Faculty of Health & Medicine Research Ethics Committee (ref FHM-2025-5527-RECR-1).
